# A pilot project to explore the mental health and wellbeing among cardiothoracic staff and the impact of virtual reality guided mindfulness

**DOI:** 10.1186/s13019-024-03089-9

**Published:** 2024-10-01

**Authors:** Bhuvaneswari Krishnamoorthy, Shabnam M. Sagar, Aman S. Coonar, Sam Raaj, Akhash V. Rathinam, Rick Air, Sarah Murray, Vanessa Heaslip, Heather Iles-Smith, Narain Moorjani, Sridhar Rathinam

**Affiliations:** 1 Directorate of Nursing and Midwifery, The University of Salford, Manchester, UK; 2 Society for Cardiothoracic Surgery in Great Britain and Ireland, London, UK; 3 Directorate of Nursing and Midwifery, University of Essex, Southend-on-Sea, UK; 4 Department of Cardiothoracic surgery, Royal Papworth Hospital, Cambridge, UK; 5 Faculty of Medicine, Imperial College London, Medical School, London, UK; 6 Faculty of Medicine, King’s College London, Medical School, London, UK; 7 Directorate of Social Science, University of Stavanger, Stavanger, Norway; 8 Department of Thoracic surgery, Glenfield Hospital, University Hospitals of Leicester, Leicester, UK

**Keywords:** Mental health and wellbeing, Stress, Anxiety, Virtual reality, Cardiothoracic surgery

## Abstract

**Background:**

The Cardio-Thoracic (CT) professional group experienced a significant increase in stress and workload during and after the COVID-19 pandemic. The Society for Cardiothoracic Surgery (SCTS) in Great Britain and Ireland with the aim of endorsing positive change. Aim of this project was to understand the Mental Health (MH) and wellbeing status of the CT professionals and to explore Virtual Reality Mindfulness as an intervention to improve MH and wellbeing.

**Methods:**

In February 2022, the SCTS created a Mental Health and Wellbeing Working Group to identify the problem and find solutions. This exploratory project was carried out in two stages. Stage one was an online survey conducted in March 2022 and stage two was a Virtual Reality (VR) mindfulness workshop in March 2023, using the Rescape™ VR mindfulness tool.

**Results:**

Stage one: An online QR code survey was sent out to 150 members with 129 (86%) completed responses. 92% expressed that SCTS should create awareness about mental health and wellbeing. 99% said that they should be allowed to speak up and create interventions for members to access, support and relax. Three main themes identified about why CT staff do not discuss their Mental Health problems were fear of lack of awareness (72%), lack of confidentiality (60%) and impact on career (60%). Stage two: 88 members attended the VR session of which 76 (86%) completed the anonymous questionnaire. 97% reported usage was a pleasurable experience, 91% felt more relaxed, 82% felt less stressed, 90% felt calmer and 89% had their mood enhanced.

**Conclusion:**

Our study findings indicate that CT staff experience considerable effects on their mental health and wellbeing. However, there is a hesitancy to recognise and seek assistance due to concerns about confidentiality and career repercussions. The virtual reality mindfulness session served as a beneficial supplement, with a positive impact in this pilot cohort.

## Introduction

Mental health is a state of mental wellbeing that enables individuals to cope with their stresses, realise their abilities, work well, learn well, and contribute to society [1]. Healthcare professionals are more likely to encounter mental health problems due to various reasons, affecting their wellbeing which have a significant impact on workforce sustainability [2, 3]. This can be seen in absenteeism, decreased productivity, increased employee turnover, and an increase in accidents and injuries [4, 5]. Within National Health Service (NHS) in 2020, 51% of work-related illness was attributed to stress, depression, or anxiety at work, which is estimated to have cost 17.9 million working days [2, 6–8]. The NHS’s burgeoning workload, significant stress, and workers’ loss of control can all lead to low staff retention and morale, staff continuity and raises provider costs associated with recruitment and training new employees [6, 9]. Thus, academics and legislators should pay greater attention to the mental health issue facing healthcare practitioners [7].

The concept of subjective wellbeing is complex and encompasses motivation and engagement, self-awareness, mindfulness, and good emotions [10, 11]. The NHS has implemented several wellbeing methods to reduce the incidence of stress at work; however, their implementation is beset with several obstacles [12, 13]. Certain wellness programmes, like mindfulness or relaxation, can require learning skills that are best taught by a therapist, making them challenge to practise throughout the workday [13, 14]. It takes time, regular practice, and effort to incorporate such techniques into a daily routine [4, 14].

### Rationale for this project

The Cardio-Thoracic (CT) professional group were significantly affected by the COVID-pandemic like many other health professionals. The post COVID-pandemic workload increased tremendously with staff shortages, low staff retention, increased complexity of cases and shortage of beds [15–18]. SCTS was aware of members’ struggle with mental health and stress and explored options to support members with interventions and support mechanisms to assist with their mental health and wellbeing. In February 2022, the SCTS set up Mental Health and Wellbeing working group to identify the problem and find the solutions.

### Aims and objectives

The aim of this pilot project was to understand the status of Mental Health (MH) of the CT professionals and introduce intervention to improve MH and wellbeing of our members, with the following objectives:

- To understand the mental health status of the CT professionals.
- To set up various mental health and wellbeing toolkits on our website and easy signposting for members.
- To introduce the latest technology of VR mindfulness workshops for members to assess whether it improves their Mental Health and Wellbeing.
- To obtain pre and post VR mindfulness workshops feedback to assess its impact on members.

### Project stages

This project was carried out in two stages. Stage one was an online survey conducted in March 2022 at the annual meeting to understand the present challenges in mental health through a survey and stage two was a Virtual Reality (VR) mindfulness workshop with a focus on the receptiveness of CT professionals to VR guided mindfulness intervention which was conducted in March 2023.

## Methods

### Ethical considerations and governance

Both stages were designed and conducted as part of our associations’ charitable members wellbeing purpose with no financial gain or profits. The Society of Cardiothoracic executive team gave permission to approach all the members. All the participants and faculties participated and attended on a voluntary basis with no contractual agreements. All the participants provided online consent to complete the survey and to attend the face to face VR sessions. Online consent was also obtained to publish the results of the survey and VR sessions feedback with no details of personal identifications.

To protect the details of all the participants the questionnaires were designed with no personal identifications in accordance with the Data Protection Act and General Data Protection Regulation guidelines (2018), all information was handled by the lead author (BK). There is no direct relationship with, nor has there been any funding received from, the Rescape™ company.

### Stage one: membership survey

The main aim of stage one was to survey members with an online tool with six themed questions which was advertised at the SCTS annual meeting in March 2022 to its members to participate voluntarily. The questionnaire was set up using Microsoft forms online with a QR code and it was completely anonymous with no identifiable data. After obtaining the verbal approval at the registration desk during the March 2022 annual meeting, the online forms were sent to 150 delegates who had agreed to participate by our administrators via QR code link of which 129 participants completed the questionnaires. The participants completed online consent to participate in this study.

### Stage two: virtual reality mindfulness session

The aim of this session was to address the issues raised by the SCTS members during the survey March 2022. The primary objectives were to understand the mental wellbeing state of members who attended the VR workshop session and their pre and post VR experience. Secondary objectives were to understand their acceptance of VR and the impact of using VR mindfulness and wellbeing session.

The VR workshop was offered as a voluntary attendance session during the annual meeting of the SCTS in 2023 in Birmingham. The team provided repeated sessions for three days to increase the opportunity for conference participants to attend and experience the VR mindfulness. There were three options given for the participants to choose from sessions lasting 3 min, 7.5 min and 15 min. No identifiable data was collected during this process except their gender and professional status. The participants were given an iPad with QR code to complete the survey pre and post VR session.

### VR experience questionnaires

The participants were asked about their state of mind, impact of VR mindfulness and the effect of the VR session on a structured questionnaire (Table 1). Four questions were asked about pre-VR mental and health wellbeing, five questions about the use of VR and selfcare post-VR session. The participants were asked to complete the pre-questionnaire before taking part in the VR-session. The questionnaires were adapted from the evaluation report of the Dr.VR^®^ Virtual Reality Headset pilot study conducted by the University Hospitals of Bristol and Weston in July 2022, for which permission was granted. The questionnaire was developed with the help of the lay members and stakeholders of the SCTS team. The proposal of this workshop was to approach the participants regarding MH and wellbeing and the survey was approved by the SCTS association executive team. All the VR sessions were set up and provided by the Rescape™ with a clinical product specialist in attendance. The workshop was advertised to the conference participants via social media and conference app.

**Table 1:** Pre and post virtual reality questionnaires

Numbers	Questions
1 (pre)	Please tell us how you felt today before starting the VR mindfulness and wellbeing session?
2 (pre)	Please tell us on a scale of 0–3, how stressed are you before starting the VR mindfulness and wellbeing session?
3 (pre)	Please tell us, before using the VR mindfulness session: how much time do you allocate to prioritise self-care/looking after yourself?
4 (pre)	Please tell us on a scale of 0–3, how calm you are before using the VR mindfulness and wellbeing session?
5 (post)	Please indicate how much you agree with the following statement: “Using the VR mindfulness and wellbeing session was a pleasurable experience”.
6 (post)	Please indicate how much you agree with the following statement: " After using the VR mindfulness and wellbeing session, I felt relaxed”.
7 (post)	Please indicate how much you agree with the following statement, “After using the VR mindfulness and wellbeing session, I felt less stressed compared to before attending the session”.
8 (post)	Please indicate how much you agree with the following statement: “After using the VR mindfulness and wellbeing session I felt calmer”.
9 (post)	Please indicate how much you agree with the following statement: “Using the VR headset enhanced my mood”.
10 (post)	Please indicate how much you agree with the following statement: “Using the VR mindfulness and wellbeing session made me to think about doing more to prioritise self-care/looking after myself.”
11 (post)	Please indicate how much you agree with the following statement: “I am happy to see that SCTS is taking time to promote delegates mindfulness and wellbeing seriously as part of the SCTS annual meeting.”
12 (post)	How likely are you to recommend this VR mindfulness session to your colleagues?

### Virtual reality set up

During each VR session, standard VR systems headsets were used or multi-projected environments to generate realistic sensations that simulate a user’s physical presence in a virtual environment. The effect is commonly created by VR headsets consisting of a head-mounted display. A wraparound headset that prevented light or images from the real world to interfere with the virtual experience. The participants chose the session timings and the clinical specialist set the session in a quiet space to create a relaxing environment.

**Fig. 1:**
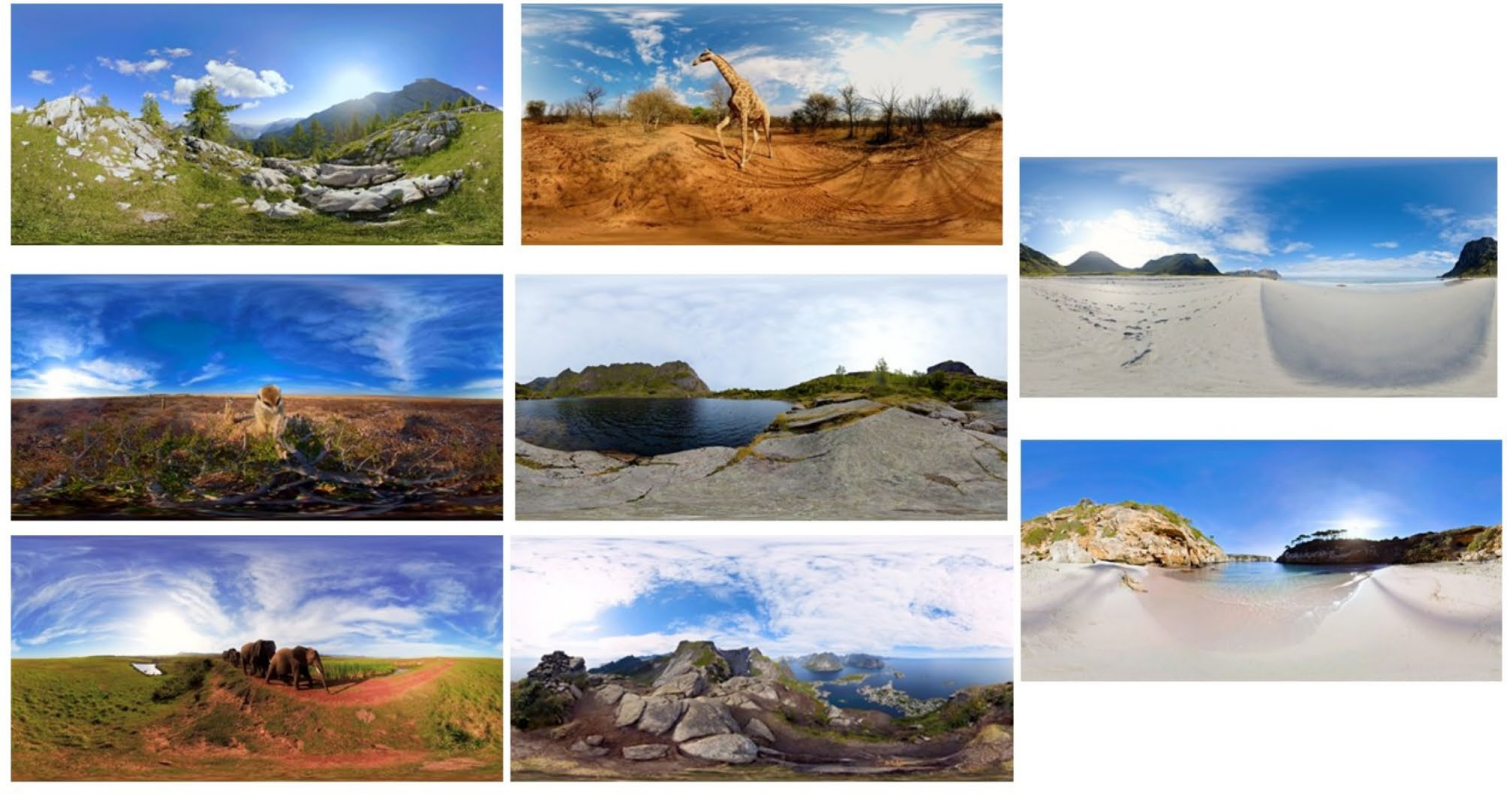
Illustrates the detailed landscape images and beautiful environment of virtual reality

Each immersive Virtual Reality experience from Rescape Innovations (https://www.rescape.health/) features the visualisation of 10 beautiful environments and landscapes (Fig. 1).

It allowed the viewer to enjoy two guided mindfulness sessions which taught breathing and relaxation techniques accompanied by the sounds of nature and soothing guidance. In between the two mindfulness sessions there was a montage of natural environments to enjoy whilst calmly relaxing and escaping to these peaceful surroundings. Members were not required to interact with this experience; they were invited to simply enjoy the experience, and to relax during the session. Participants were instructed to discontinue use if they experienced any discomfort or side effects (headache, dizziness, motion sickness, eye irritations and claustrophobia); this was recorded as a side effect and limitation of the therapy session.

## Results

### Results of stage 1 survey

129 participants completed this survey of which were 36 (28%) nurses, 30 (23%) consultant surgeons, 23 (18%) senior specialist trainee doctors, 7 (16%) junior doctors, 21 (6%) surgical care practitioners, 7 (5%) allied health professionals (5%) and 5 (4%) paediatric cardiothoracic surgeons.

95% (*n* = 123) mentioned that SCTS should do more mental health webinars, seminars to promote wellbeing of their members. 92% (*n* = 119) said that SCTS should create better awareness in mental health and wellbeing and provide materials on the website for members to access and get support from professional groups. 99% (*n* = 127) said that they should be allowed to speak up, provide case studies, podcasts on mental health and wellbeing and interventions for members to access, support and relax.

**Fig. 2:**
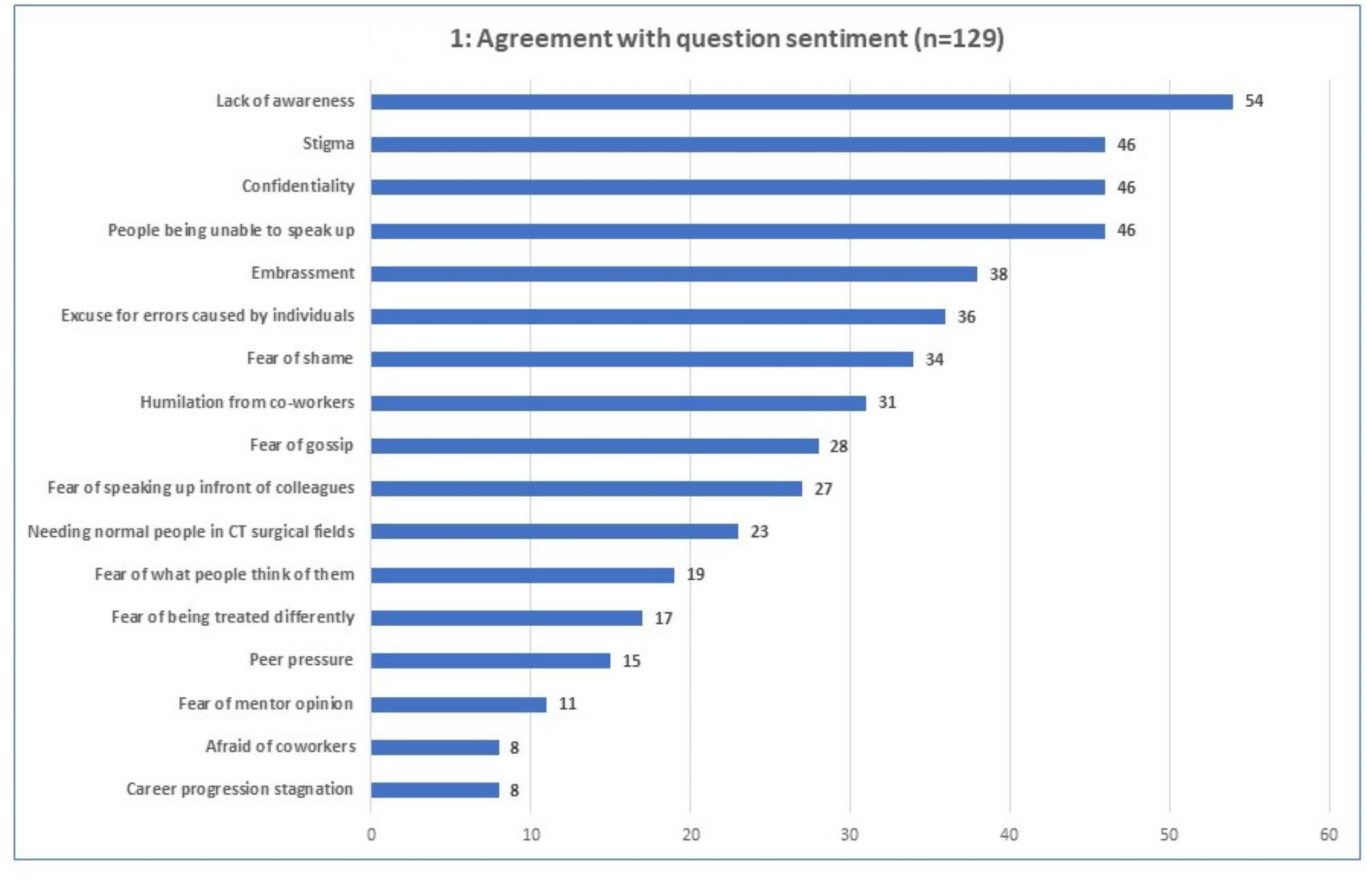
Illustrates the members reasoning not to talk about mental health issues

In addition to above mentioned categories, each member who completed the survey was asked to state three reasons that prevent cardiothoracic staff from opening up about their mental health problems. Their responses were themed into 17 themes for clarity on Fig. 2.

### Impact of survey results

The results of the survey were presented at the European Cardiothoracic Surgery Annual Meeting in Milan 2022. The SCTS working group realised the importance of supporting the members. SCTS developed a dedicated website support and mental health wellbeing sessions such as Yoga, Tai Chi and Virtual Reality mindfulness and wellbeing sessions during most of the educational events and annual meeting held in 2023/2024. The society also offered members support from the SCTS officers, lay member as well as regional board of representatives and mentors who could support members with issues pertaining to mental health and well-being. The SCTS also offered links to non-profit organisations which offered support to medical and allied health professionals with regards to mental well-being.

### Results of stage 2 VR session

VR sessions were conducted for 3 days to capture as many volunteers as possible who attended the SCTS annual meeting in 2023. A total of 88 volunteers attended the VR session and 76 completed the anonymous questionnaire. 95% underwent the 7.5 min and 5% chose the 3 min session. Regrettably, the 15-minute session went unselected as attendees had prior commitments during the SCTS annual conference.

66% participants were female in the workshop. The participants engagement with the session was well distributed over the three days, 19th (38%), 20th (33%) and 21st March 2023 (29%).

Majority of the members who attended the VR session were 31 Nurses (40%), followed by 16 Consultant Surgeons (21%), 6 Allied Health Professionals (8%), 6 Advanced Clinical Practitioners (8%), 8 Specialist Registrars (10%), 2 Medical students (3%), 6 Research fellows (8%) and 1 Operating Department Practitioners (2%).

The participants were asked their state of mind, impact of the VR simulation and the effect of the VR simulation exercises on a structured questionnaire (Table 1).

### Pre-virtual reality questionnaire

1. Participants responded that they felt anxious (49%), neutral (26%), excited (11%), calm (9%) and annoyed (5%) prior to the session.
2. On evaluation about stress, 17% expressed they were not stressed at all, 56% were somewhat stressed, 21% were moderately stressed while 6% were severely stressed.
3. Whilst exploring time that the participants allocate to prioritise self-care/looking after themselves before using the VR mindfulness session: 7% participants stated they never allocated time for self-care/looking after themselves, 10% devoted at least 1 h every day for themselves, 4% participants devoted 2 h for themselves, 3% dedicated for 3 h, and another 3% used half a day for themselves, 32% spent once in a week,10% take care of themselves twice a week and 21% only once a month.
4. The study explored their state of calm before the session: 7% participants responded they were not calm before using VR mindfulness and wellbeing session, 47% participants experienced mild relaxation, 41% felt moderately calmed whereas 5% participants were severely calm.

**Fig. 3:**
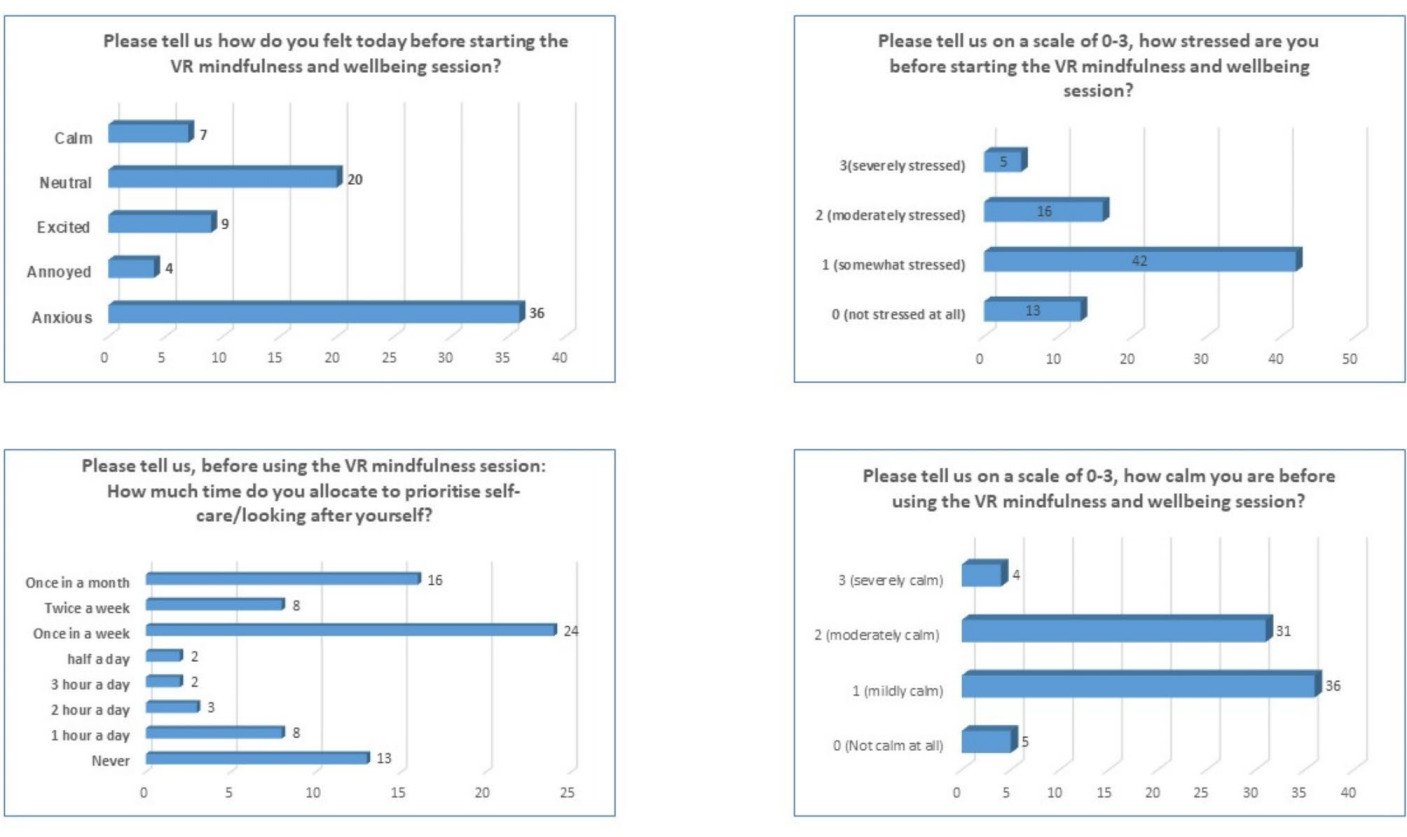
Illustrates the summary of results of pre-virtual reality questionnaire

The results are summarised in Fig. 3.

### Impact of virtual reality experience

1. 96% of users stated that using the VR mindfulness and wellbeing session was a pleasurable experience, followed by 3% neither agreed nor disagreed and 1% strongly agreed.
2. 92% of respondents agreed after using the VR mindfulness and wellbeing session, they felt relaxed, followed by 6% who strongly agreed and 2% who neither agreed nor disagreed.
3. 86% felt less stressed than before attending the session, followed by 7% who strongly agreed and 7% who neither agreed nor disagreed.
4. 86% agreed that they felt calmer after using the VR mindfulness and wellbeing session with 11% strongly agreeing and 3% neither agreed nor disagreed.
5. 96% agreed that using the VR headset enhanced their mood, followed by 3% strongly agreed and 2% neither agreed nor disagreed.
6. 86% agreed that participating the VR mindfulness session stimulated them to think about prioritising self-care, followed by 7% who strongly agreed and 7% neither agreed nor disagreed.
7. 96% mentioned very likely to recommend the VR mindfulness session to their colleagues, followed by 4% who stated that it was somewhat likely.

**Fig. 4:**
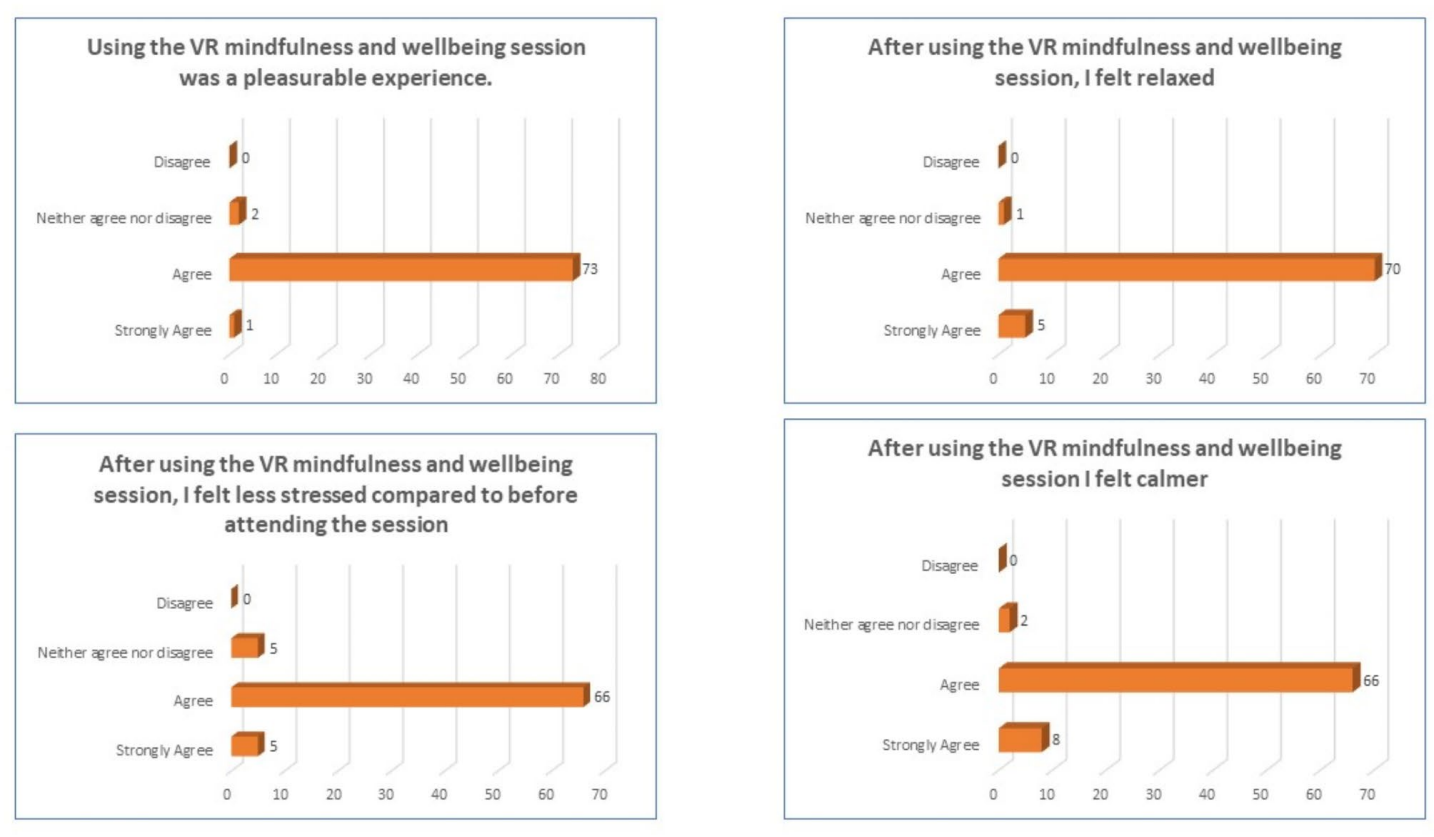
Illustrates the summary of results of post-virtual reality questionnaire

These results are summarised in Fig. 4.

### General comments about VR session

We did ask the participants to leave generic comments. 46% mentioned that they enjoyed it a lot, 24% said it was fun, cool and exciting technology, 20% said it should be available for staff and patients in the NHS, 4% said that people are crazy nowadays, VR session made me relaxed, 4% said that it is great to see wellbeing and mindfulness being explored in an virtual reality environment and the enclosed headset is a great idea, 2% said that they felt relaxed, but they didn’t like the claustrophobic feeling of using the VR headset.

## Discussion

Our two-stage project validated the impact of a stressful environment on the mental health and wellbeing of the cardiothoracic workforce. The survey results highlighted the reasons and rationale on why the participants do not acknowledge that they have mental health problems because of the lack of awareness (72%). The fact it is perceived as a social stigma (70%) on which people will judge them or have peer judging (20%) or result in challenges in career progression (10%) as our participants acknowledge compounds the problem. This was highlighted on an opinion article by Palethorpe [19] that 99% trust leaders were extremely or moderately concerned about the resilience and wellbeing of their staff. Importantly, they insisted that the negative effect of burnout is seen not only in health care professionals’ wellbeing but also in clinical outcomes [20].

It is imperative that providers and employers acknowledge the importance of mental wellbeing and look at various options to help the workforce of which the virtual reality mindfulness tool is a unique option [21].

Our pilot study results reinforce the growing body of literature supporting the proposition that VR can be an effective tool for the promotion of relaxation and the reduction of stress at non-clinical area [22]. The participants self-reporting questionnaire results identified that the DR. VR^®^ pilot was very well received and had a positive impact on participants who were able to engage in the voluntary one-off VR sessions. Similarly, Fincham et al., reported that practicing mindfulness for just 10–30 min can yield similar positive results on mental wellbeing, but their participants were instructed to practice daily for two weeks [23]. Another study found that the use of VR with NHS staff in a fast-paced work environment enhances their satisfaction and improves their urge to practice mindfulness as part of their daily routine activities [24].

Following the VR session, the participants expressed feeling more at ease and optimistic. Many also stated that they believed having frequent access to this technology during the working day would improve their general well-being at work. Similar results were found by Naylor et al., (2019) that Virtual Reality can offer wellbeing interventions that are exceptionally immersive also that engaging and participatory virtual reality experiences could promote calmness and less stress [25]. Some of the general comments left by the participants in form of free text supported the quantitative results and provided a deeper understanding of participants thoughts and feelings about the VR mindfulness session experiences.

The Virtual Reality mindfulness session is at the forefront of technological innovations to improve the mental health and wellbeing. The head-mounted display technology, which provides accessible means of promoting relaxation through visualisation, engagement, and immersion with enjoyable virtual surroundings, is commonly referred to as “VR” [22]. The VR users are taken out of stressful situations by entering tranquil virtual audio-visual settings, which promotes relaxation and stress management in the face of daily obstacles [26]. Some of the recent studies demonstrated that pleasant virtual environments could lead to complete relaxation and decrease tension, and anxiety [26–28].

The NHS is the largest employer in England, with around 1.1 million full-time equivalent staff in hospital and community services and its workforce constitutes the system’s greatest asset [24]. So, it is vital that we protect our staff and provide mental health and well-being support to reduce work-related stress and anxiety.

There remains a gap in the literature in maintaining the health and well-being of the UK population and disproportionately affected by workforce stress, particular attention to promote their overall health and well-being is warranted. No studies assessing the efficacy of VR in this area have focused on frontline healthcare professionals within the NHS. Given that NHS staff are an integral part of the workforce, and it is imperative that we explore all options to ensure staff health and wellbeing.

### Limitations

The study has several limitations due to the nature of the survey data collection during the SCTS annual meeting. One of the main limitations was that it was a voluntary participation rather than research enrolment. There was no control group to which the VR intervention could be compared and thus, a causal relationship between the VR experiences of the participants could not be established. The second limitation was that the mental status or pre-existing mental health issues of the participants were not identified which would have helped to understand the true impact of the VR mindfulness and wellbeing of the CT professionals. Our focus to get funding for the VR session study at workplace to get more in-depth information from the participants, due to the limitations of the SCTS meeting programme, we provided single VR session for the participants as a taster session which is another limitation of this project.

## Conclusion

Mental health wellbeing and its impact as well as the reasoning of cardiothoracic workforce choosing not to disclose was evident in the study. It highlighted the need for engagement and support in establishing mental health and wellbeing in the fraternity. The result of the study clearly suggests that the VR interventions to be promising in workforce contexts given the small number of studies that focus on VR for promoting mental health and wellbeing in the workplace.

## Data Availability

The data will be available on request. All the coded data is stored with the lead author, and we are happy to provide anytime on request.

## Declarations

Human Ethics and Consent to participate declarations: Nothing to declare.
